# Development of a Novel Biosensor Based on Tyrosinase/Platinum Nanoparticles/Chitosan/Graphene Nanostructured Layer with Applicability in Bioanalysis

**DOI:** 10.3390/ma12071009

**Published:** 2019-03-27

**Authors:** Irina Mirela Apetrei, Constantin Apetrei

**Affiliations:** 1 Department of Pharmaceutical Sciences, Medical and Pharmaceutical Research Center, Faculty of Medicine and Pharmacy, “Dunarea de Jos” University of Galati, 800008 Galati, Romania; irina.apetrei@ugal.ro; 2 Department of Chemistry, Physics and Environment, The European Centre of Excellence for the Environment, Faculty of Sciences and Environment, “Dunarea de Jos” University of Galati, 800008 Galati, Romania

**Keywords:** biosensor, screen-printed carbon electrode, nanomaterial, nanoparticle, bionanocomposite, square wave voltammetry, L-tyrosine, bioanalysis

## Abstract

The present paper describes the preparation and characterization of a graphene, chitosan, platinum nanoparticles and tyrosinase-based bionanocomposite film deposited on the surface of a screen-printed carbon electrode for the detection of L-tyrosine by voltammetry. The redox process on the biosensor surface is associated with the enzymatic oxidation of L-tyrosine, which is favoured by graphene and platinum nanoparticles that increase electrical conductivity and the electron transfer rate. Chitosan ensures the biocompatibility between the tyrosinase enzyme and the solid matrix, as well as a series of complex interactions for an efficient immobilization of the biocatalyst. Experimental conditions were optimized so that the analytical performances of the biosensor were maximal for L-tyrosine detection. By using square wave voltammetry as the detection method, a very low detection limit (4.75 × 10^−8^ M), a vast linearity domain (0.1–100 μM) and a high affinity of the enzyme for the substrate (K_M_^app^ is 53.4 μM) were obtained. The repeatability of the voltammetric response, the stability, and the reduced interference of the chemical species present in the sample prove that this biosensor is an excellent tool to be used in bioanalysis. L-tyrosine detection in medical and pharmaceutical samples was performed with very good results, the analytical recovery values obtained being between 99.5% and 101%. The analytical method based on biosensor was validated by the standard method of analysis, the differences observed being statistically insignificant at the 99% confidence level.

## 1. Introduction

The recent development of sensors and biosensors based on nanomaterials for detecting amino acids which are vital for an organism, such as L-Tyrosine (Tyr), is of utmost interest in bioassay [1]. L-Tyrosine is one of the indispensable amino acids found in proteins, which is necessary in order to maintain a positive nitrogen balance in the body [2,3]. Tyr is the precursor of a series of compounds such as thyroxine, dopamine, epinephrine and norepinephrine, which condition the appropriate functioning of the body [4]. Tyr can be synthesized by the body if a sufficient amount of phenylalanine is given by exogenous intake [5]. The Tyr level in the body is correlated with the individual’s health and its normal concentration level in blood plasma ranging between 30–120 μM [6].

The presence of this amino acid in small amounts may cause a range of diseases such as depression, hypochondria and physical/mental exhaustion, its absence possibly leading to albinism and alkaptonuria. On the other hand, high levels of Tyr in blood are due to the deficiency of enzymes such as tyrosine aminotransferase in the Tyr catabolic pathway. This deficiency favours metabolic diseases such as tyrosinemia and hypertyrosinemia, whose symptoms are poor liver and kidney function and intellectual disability. Therefore, since the Tyr concentration in the human body may be an indicator of health status, it needs to be determined quickly and precisely [7,8]. 

Up to the present time, various analytical methods such as spectrometry [9], chemiluminescence [10], capillary electrophoresis [11], high performance liquid chromatography coupled with mass spectrometry [12], and electrochemical methods [8,13] have been used for determining Tyr in biological fluids.

In spite of the wide range of practical tools available, the use of these analytical methods is limited by some drawbacks such as laborious sample preparation, long analysis time and high costs [8]. On the other hand, electrochemical methods used for the detection of numerous compounds useful in biomedical analysis are simple, fast and cost-effective [14]. Determination of amino acids by means of solid electrodes is difficult due to their low electroactivity. Therefore, a chemical modification of the electrodes is required to improve their electrochemical response [13]. The most important drawbacks of these chemically modified electrodes are the contamination of the active surface and reduced selectivity [15]. These problems may be solved by immobilizing an appropriate enzyme at the surface of the electrode and by using nanomaterials in order to increase sensitivity and selectivity.

Tyrosinase is one of the most commonly used enzymes for detecting phenolic compounds from different types of samples. It is a type III copper protein found in various fungi, plants and mammals which is activated by the binding of an oxygen molecule and which may act as both mono-phenolase and diphenolase [16].

Graphene (GPH) is a monolayer of sp^2^ hybridized carbon atoms, which are bound in a hexagonal planar system. This nanomaterial has a range of physicochemical, electronic, mechanical, and thermal properties superior to other materials and it is used in electronics, batteries, combustion cells, supercapacitors and biosensors [17,18]. On the other hand, platinum nanoparticles have an electrocatalytic effect and are used to develop different types of sensors and biosensors [19,20]. Chitosan is a natural biopolymer with properties such as biocompatibility, thin film deposition, water permeability, and high mechanical strength which are suitable for building a solid matrix in which the enzyme has to be immobilized. Chitosan is compatible with both enzymes, metallic nanoparticles and carbon-based materials and may facilitate enzymatic reactions and electron exchange towards the biosensor interface [19,21]. 

Until now, tyrosinase immobilized in different nanostructured films has been extensively used in the biosensors development. For example, Rahimi-Mohseni and co-workers proposed a new tyrosine biosensor using the co-catalytic effect of tyrosinase from banana peel tissue (*Musa* Cavendish) and functionalized silica nanoparticles immobilized on graphite screen-printed electrodes [8]. In another study, Carralero and co-workers reported a high analytical performance-tyrosinase biosensor based on a composite graphite-teflon electrode modified with gold nanoparticles for detecting of different phenolic compounds. The presence of gold nanoparticles enhances the kinetics of the reactions involved in the biochemical recognition process and in the electrochemical transduction [22]. Yang and co-workers developed a new nanocomposite film of tyrosinase-chitosan-carbon-coated nickel nanoparticles for the detection of catechol. The immobilization of tyrosinase on the chitosan-carbon-coated nickel nanoparticles film was favourable, and tyrosinase retained its bioactivity to a large extent [23]. An electrochemical biosensor for phenol derivatives based on the covalent bonding of tyrosinase onto a graphene oxide-modified glassy carbon electrode via glutaraldehyde was reported. In this case, the hydroxyl group on the graphene oxide was useful for the covalent coupling of enzyme to the surface [24]. Singh et al. presented the synthesis and application of polypyrrole and gold nanoparticles film and the immobilization of tyrosinase enzyme for tyrosine and catechin biosensing. The polypyrrole acts as conducting matrix and gold nanoparticles play the role of electrocatalysts [25]. 

The graphene, platinum nanoparticles, chitosan were also used for the immobilization of other enzymes in the sensitive element of different biosensors with enhanced performance characteristics. For instance, a biosensor based on diamine oxidase/platinum nanoparticles/graphene/chitosan modified screen-printed carbon electrode showed good performance in the electrochemical detection of histamine. Enhanced sensitivity is related to the electrocatalytic synergetic effect of graphene and platinum nanoparticles on the electrochemical detection of H_2_O_2_ [26]. Another biosensor based on platinum nanoparticles-reduced graphene oxide-laccase biocomposite for the determination of total polyphenolic content was developed. The combination of reduced graphene oxide and platinum nanoparticles leads to a synergistic effect, increasing the electroactive surface area of the electrode and enhancing electron transfer towards the electrode [27]. Therefore, the development of a composite nanomaterial based on graphene, platinum nanoparticles, chitosan and tyrosinase in order to build a new biosensor with superior characteristics is a complex and challenging research task.

The present paper describes the development of a new biosensor for detecting tyrosine, which has applicability in bioanalysis. The biosensor was fully characterized and experimental conditions were optimized so that sensitivity and selectivity were maximal. The analytical method based on the biosensor was validated in the laboratory by using the standard method for determining tyrosine.

## 2. Materials and Methods

### 2.1. Reagents

L-Tyrosine (L-2-Amino-3-(4-hydroxyphenyl)propanoic acid, 4-hydroxyphenylalanine), Tyrosinase (from mushroom, EC 232-653-4, activity 5370 U/mg of solid), chitosan, acetic acid, hexachloroplatinic acid (H_2_PtCl_6_), sulphuric acid (H_2_SO_4_), sodium phosphate monobasic (NaH_2_PO_4_), sodium phosphate dibasic (Na_2_HPO_4_), phosphoric acid (H_3_PO_4_), sodium hydroxide (NaOH), sodium chloride (NaCl), potassium chloride (KCl), calcium chloride (CaCl_2_), magnesium chloride (MgCl_2_), glycine, L-lysine, L-asparagine, L-Phenylalanine, reduced L-glutathione, uric acid were purchased from Sigma-Aldrich (Saint Louis, MO, USA). d-(+)-glucose (Acros Organics, NJ, USA) and L-ascorbic acid (Riedel-de Haën, Seelze, Germany) were also purchased and used as received. All the solutions were prepared in ultrapure water (obtained in a Milli-Q Simplicity^®^ Water Purification System, Merk, Darmstadt, Germany).

### 2.2. Materials

Graphene (GPH) powder, with electrical conductivity >10^3^ S·m^−1^ and surface area >500 m^2^ g^−1^ (BET—Brunauer-Emmett-Teller) from Sigma-Aldrich (Saint Louis, MO, USA) was used for the modification of carbon screen-printed electrode (CSPE). Carbon screen-printed electrodes (working electrode from C, auxiliary electrode from C, reference electrode from Ag) were purchased from Metrohm-Dropsens (Llanera, Spain). CSPEs were successively modified with GPH, Chitosan (Chit), Pt nanoparticles (PtNP) and tyrosinase (Ty) in order to get a novel enzymatic nanostructured biosensor.

### 2.3. Development of Platinum Nanoparticles/Chitosan/Graphene-Carbon Screen-Printed Electrode (PtNP/Chit/GPH-CSPE)

The dispersion of GPH was prepared by mixing 1 mg GPH with 1 mL chitosan solution (0.2% in acetic acid, pH = 5) followed by ultrasonication for 2 h. By this method a homogeneous dispersion of GPH in aqueous phase was obtained. CSPE was modified with 10 μL GPH dispersion by the casting method. The evaporation of the solvent was carried at room temperature in a desiccant. 

After drying, on the surface of GPH/Chit-CSPE platinum nanoparticles (PtNP) were deposited by chronoamperometry at +0.4 V for 300 s from a 2 × 10^−3^ M H_2_PtCl_6_ and H_2_SO_4_ aqueous solution. The counter electrode was a Pt plate of 2 cm^2^ and Ag/AgCl, KCl (3.5M) was the reference electrode. The PtNP/GPH/Chit/CSPE was rinsed with ultrapure water and dried in a desiccant [19,20].

### 2.4. Development of Tyrosinase (Ty)/PtNP/GPH/Chit-CSPE

The tyrosinase enzyme was immobilized on the surface of PtNP/GPH/Chit-CSPE by the casting method. Thus, 10 μL of 0.1 M phosphate-buffered saline (PBS) (pH = 7) containing 50 μg/μL of tyrosinase was added onto the surface of PtNP/GPH/Chit-CSPE. The biosensor was dried at room temperature in a desiccant overnight. The Ty/PtNP/GPH/Chit-CSPE biosensors were kept in a closed box at 4 °C in a fridge in order to prevent the denaturation of the sensitive element. 

### 2.5. Apparatus

All the electrochemical results obtained from voltammetric experiments (cyclic voltammetry and square wave voltammetry) were recorded by a Biologic SP 150 potentiostat/galvanostat (Bio-Logic Science Instruments SAS, Claix, France) controlled by the EC-Lab Express software V5.52. All measurements were carried out at room temperature, ambient conditions and atmospheric pressure. The scanning electron microscope (SEM) images were captured by a FlexSEM 1000 (Hitachi, Tokyo, Japan) scanning electron microscope. A Cencom II centrifuge (JP SELECTA S.A., Barcelona, Spain) was used to centrifuging the sample solutions. An Inolab pH 7310 pH-meter (WTW, Weilheim, Germany) equipped with a combined glass electrode/Ag/AgCl was applied to pH buffer solutions adjustments. The ultraviolet (UV)spectrometric experiments were carried out with a Rayleigh UV-1601 spectrophotometer (Beijing Rayleigh Analytical Instrument Corporation, Beijing, China).

### 2.6. Biological and Pharmaceutical Samples

The determination of Tyr was carried out in heparinized blood plasma supplied by one hospital laboratory. The pharmaceutical samples were purchased from local pharmacies. All subjects gave their informed consent for inclusion before they participated in the study. The study was conducted in accordance with the Declaration of Helsinki, and the protocol was approved by the Ethics Committee of “Dunarea de Jos” University of Galati (#3158/17). A recovery study was carried out by by spiking different known concentrations of the Tyr solution. Three replicate measurements (n = 3) were carried out for each measurement. The Tyr concentration was calculated from the calibration curve by employing the corresponding dilution factor.

## 3. Results

### 3.1. Characterization of Nanostructured Biomaterial 

**Figure 1:**
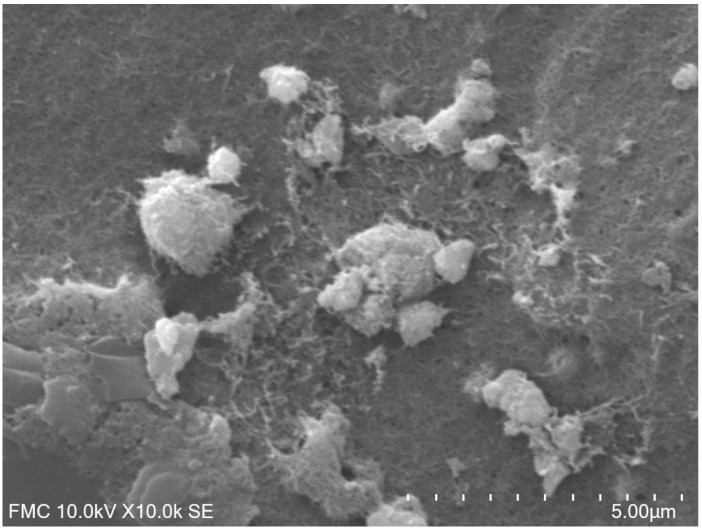
Scanning electron microscope (SEM) image of CSPEmodified with tyrosinase/platinum nanoparticles/graphene/chitosan (Ty/PtNP/GPH/Chit) bionanocomposite.

Figure 1 shows the SEM image of the Ty/PtNP/GPH/Chit-CSPE, which presents a typical 3D morphology, confirming the attachment of the GPH on the surface of CSPE, the successful PtNPs electrodeposition and the immobilization of tyrosinase enzyme.

### 3.2. Exploratory Studies for the Detection of Tyr by Biosensor

**Figure 2:**
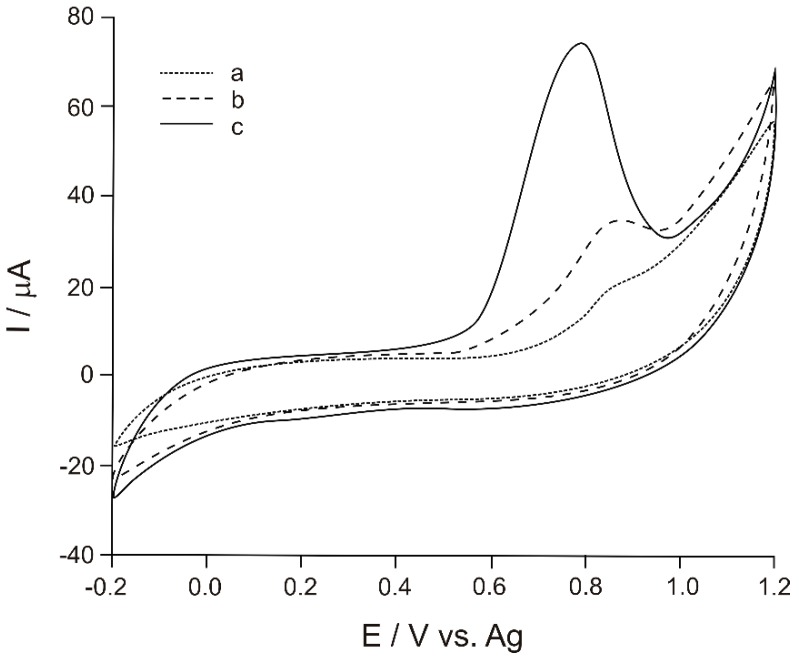
Cyclic voltammograms (CVs) of CSPE (curve a), PtNP/GPH/Chit-carbon screen-printed electrode (CSPE) (curve b), Ty/PtNP/GPH/Chit-CSPE (curve c) immersed in 10^−4^ Tyr, PBS 0.1M, pH = 7.

Figure 2 illustrates the cyclic voltammograms (CVs) of CSPE (curve a) recorded in a 10^−4^ M Tyr solution (0.1 M phosphate buffer, pH = 7) versus the pseudo-reference Ag electrode. As it may be noticed in Figure 2, all modified electrodes have a higher anodic peak and lower peak potentials as compared to the unmodified CSPE.

The best results were obtained by the biosensor (Ty/PtNP/GPH/Chit-CSPE) (curve c), which has the highest peak current (74 μA) and the lowest potential corresponding to this peak (0.79 V). The peak current identified for Ty/PtNP/GPH/Chit-CSPE is ≈4 times higher than that of CSPE (18.4 μA) and the anode peak potential is about 70 mV more reduced as compared to the positive potential observed for CSPE (0.86 V). The differences between the biosensor and the PtNP/GPH/Chit-CSPE electrode (curve b) are important, as well, the peak current being 2 times higher and the potential being shifted to the lower potential with 90 mV.

These results prove that Ty immobilized in the matrix of nanostructured materials is viable and acts efficiently on the oxidation of the substrate.

**Figure 3:**
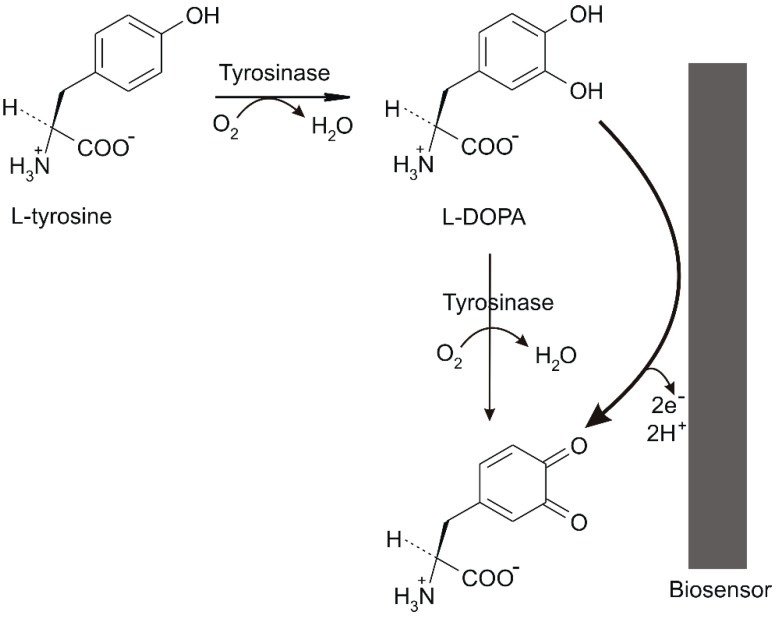
Biosensor detection mechanism.

The mechanism of the biosensor detection is illustrated in Figure 3.

Tyrosinase catalyses the Tyr hydroxylation reaction in the ortho position relative to the hydroxyl group (cathecholase) by transforming it into the L-3,4-dihydroxyphenylalanine (levodopa, L-DOPA) amino acid. The subsequent stage is represented by the oxidizing of the ortho-quinonic derivative (a-dopaquinone) [13,28].

The biosensor detection mechanism described proves that the enzymatic reaction is dependent on the concentration of O_2_ in the solution and on the pH of the medium. Therefore, biosensor measurements should be carried out under ambient conditions in the presence of air and in buffer solution with the pH suitable for the optimal functioning of the enzyme [13,28].

By comparing the cyclic voltammograms of electrodes which were modified successively with nanomaterials or biomaterials, their influence and role in the biosensor detector element may be determined.

Furthermore, by comparing the cyclic voltammograms of PtNP/GPH/Chit-CSPE and Ty/PtNP/GPH/Chit-CSPE, the increased sensitivity of the electrode due to incorporating the enzyme into the solid matrix is obvious. The decrease of the anodic peak potential related to Tyr oxidation process in the presence of the enzyme may be noticed, as well.

When the cyclic voltammograms of CSPE, Chit/GPH-CSPE and PtNP/GPH/Chit-CSPE are compared, an increase in the anodic peak current may be observed, aspect which indicates that both GPH and PtNP facilitate the reaction from the biosensor surface. The differences regarding the mechanisms of action are significant. Thus, GPH through the bidimensional special nanostructure increases the electrical conductivity of the sensitive layer, while PtNPs facilitate the transfer of electrons to the biosensor surface [18,20]. The corresponding effects are synergistic when both nanomaterials are components of a nanocomposite material.

### 3.3. Influence of the Scan Rate in the Biosensor Response

**Figure 4:**
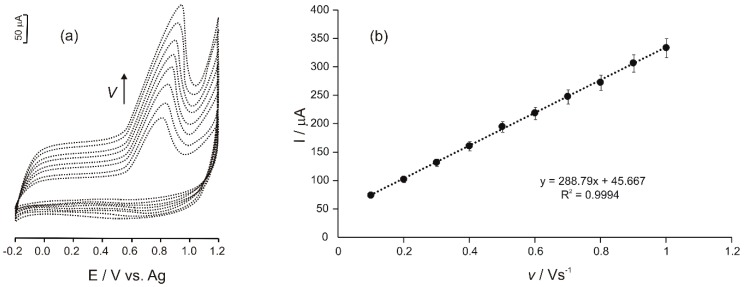
(**a**) CVs of Ty/PtNP/GPH/Chit-CSPE immersed into a Tyr solution (10^−4^ M) prepared in phosphate-buffered saline (PBS) (0.1 M, pH = 7) at different scan rates ranging between 0.1–1.0 V·s^−1^ and (**b**) variation of anodic peak current versus scan rate. Measurements were performed in triplicate (Relative standard deviation, RSD = 3.54%).

The cyclic voltammograms of Ty/PtNP/GPH/Chit-CSPE immersed in a 10^−4^ M Tyr solution (0.1 M phosphate buffer, pH = 7) were recorded at different scanning rates in the range of 0.1–1.0 V·s^−1^. The results obtained are shown in Figure 4a,b.

As it may be noticed, an increased scanning rate leads to an increase in the anodic peak current and to a slight shift of the peak potential to more positive values. Anodic peak current associated with Tyr oxidation at the surface of the biosensor varies linearly with the scanning rate (I = 288.79*v* + 45.667; R² = 0.9994), proving that the electrochemical process is controlled by the adsorption process (Figure 4b) [29].

Additionally, the heterogeneous electron transfer rate constant (*k*^0^) of PtNP/GPH/Chit-CSPE and Ty/PtNP/GPH/Chit-CSPE electrodes detecting Tyr was calculated using Kochi’s method based on Equation (1):

(1) $$k^{0}=2.18{\left(\frac{\alpha vnFD}{RT}\right)}^{1/2}EXP\left(-\frac{\alpha^{2}nF\Delta E}{RT}\right)$$

where: *α* is the electron transfer coefficient (*α* = 0.5), *n* is the number of electrons exchanged into redox process (*n* = 2), *D* is the diffusion coefficient (*D* = 3 × 10^−5^ cm^2^·s^−1^), *F* is Faraday constant (96,485 C·mol^−1^), *v* is the scan rate (*v* = 0.1 V·s^−1^), *R* is the gas constant (8.314 J·mol^−1^·K^−1^), *T* is the temperature (*T* = 293 K) [30,31,32]. 

The shift of the anodic peak in the absence and the in presence of Ty was used for calculating the heterogeneous electron transfer rate constants for PtNP/GPH/Chit-CSPE and for Ty/PtNP/GPH/Chit-CSPE electrodes. The values obtained for PtNP/GPH/Chit-CSPE and Ty/PtNP/GPH/Chit-CSPE were 4.97 × 10^−3^ cm·s^−1^ and 2.74 × 10^−3^ cm·s^−1^, respectively. The presence of Ty in the sensitive layer increased the electron transfer rate constant 1.8 times.

### 3.4. Studies for the Optimization of the Experimental Parameters

**Figure 5:**
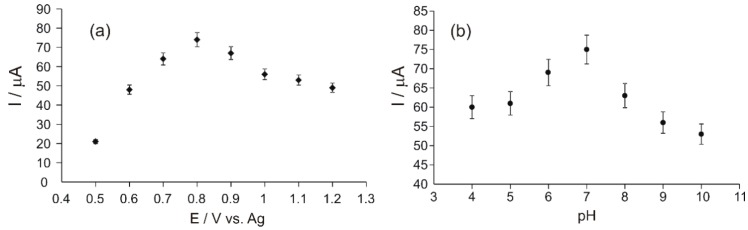
Effects of applied potential (**a**) and pH (**b**), respectively, on the response of Ty/PtNP/GPH/Chit -CSPE biosensor.

In order to obtain the optimal value of the applied potential, the amperometric signal of Ty/PtNP/GPH/Chit-CSPE towards Tyr was determined under continuous and constant stirring of the sample. The applied potential is one of the most relevant parameters, which significantly influences the electrochemical signal of a biosensor, affecting both sensitivity and selectivity. The most intense response of the biosensor was obtained at +0.8 V vs. Ag (Figure 5a). Therefore, the sensitivity of the sensor is maximum for Tyr detection when this value of the potential is applied.

pH optimization is necessary in order for the enzyme to have the best biocatalytic activity and to avoid the denaturation of the enzyme immobilized in the detector element of the biosensor. The pH influence on the electrochemical determination of L-Tyrosine with Ty/PtNP/GPH/Chit-CSPE was studied by amperometry in Tyr 10^−4^ M solutions in 0.1 M PBS with different pHs in the range from 4 to 10. The response of the Ty/PtNP/GPH/Chit-CSPE biosensor to Tyr 10^−4^ M increased when the pH was modified from 4 to 7, and decreased when the pH reached values from 7 to 10 (Figure 5b).

The decreased biosensor response may be related to the enzyme denaturation, this leading to the loss of biocatalytic activity. Based on the results of this present study, it was decided that the electrochemical determination of Tyr should be performed at the optimal pH value of 7.

### 3.5. Analytical Performance Characteristics of the Ty/PtNP/GPH/Chit-CSPE Biosensor

In order to achieve superior analytical performance, the square wave voltammetry (SWV) electroanalytical detection technique was used. This method has a much greater sensitivity as compared to cyclic voltammetry or amperometry [33].

**Figure 6:**
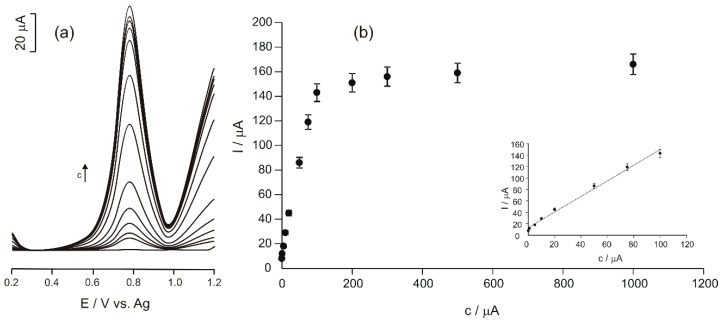
(**a**) The square wave voltammetry readings (SWVs) of Ty/PtNP/GPH/Chit-CSPE biosensor immersed in Tyr solution with different concentrations ranging from 1 × 10^−7^ to 1 × 10^−3^ M; (**b**) the dependence between the peak current values and the concentration values (calibration curve). B inset—the linearity range.

The optimal parameters of the SWV method were: pulse amplitude 0.090 V, increment 5 mV, frequency 15 Hz. The potential range used was 0.2 to 1.2 V. The voltammograms obtained in Tyr solutions of variable concentrations are shown in Figure 6.

Analysing the voltammograms obtained by using the SWV technique, the fact may be noticed that the anodic peak of Tyr is better defined as compared to that observed in the cyclic voltammograms. This result is due to the application of potential in the form of a pulse, which reduces the consumption of the electroactive species and the background current.

The relationship between the SWV responses and the concentrations of Tyr solutions was determined by using the regression method (Figure 6b). 

The dependence obtained is typical for a biosensor, a linearity range and a plateau, where the current of the anodic peak does not increase when the concentration is increased.

The linearity range between the anodic peak current and the Tyr concentration of the solutions analysed was 0.1–100 μM, as it may be noticed in Figure 6b (insert).

The limit of detection (LOD) of the biosensor developed in this study was calculated in accordance with the IUPAC recommendations (3 S/m, where S is the standard deviation of the blank, and m is the slope of the calibration curve) [34]. The replicates number of the blank measurements for the S calculation was 7. The LOD of the biosensor for L-tyrosine in the range of 0.1–100 μM was found to be 4.75 × 10^−8^ M.

The Hill coefficient was determined from the dependence equation between log[*I*/(*I_max_* − *I*)] vs. log [*Tyr*], which is the slope of this dependence. The value of the Hill coefficient was 1.02, a value very close to the ideal value, which suggests that the enzymatic reaction follows a type of Michaelis–Menten mechanism [35]. 

Moreover, the biosensor calibration curve and the steady-state current mathematical expression of the Lineweaver-Burk equation for an electrochemical system (Equation (2)) were used to calculate the enzyme kinetics parameters [36].

(2) $$I=\frac{I_{max}\left[Tyr\right]}{\left[Tyr\right]+K_{M}^{app}}$$

where: *I* is the anodic current, *I_max_* is the steady-state current, [*Tyr*] is the molar concentration of tyrosine, and $K_{M}^{app}$ is the apparent Michaelis–Menten constant.

The apparent Michaelis-Menten constant calculated for the immobilized tyrosinase was 53.4 μM, a value lower than other values reported in the literature, indicating the efficient immobilization of the enzyme in the bionanocomposite material [26,29,37].

The performance characteristics of the biosensor recommend it for the applications in the analysis of Tyr in real samples.

### 3.6. A Comparative Approach to the (Bio)Sensors Reported in the Literature

The results obtained by using the biosensor developed in this research study (linear range, LOD) were compared to a series of results reported in literature and the results obtained are shown in Table 1.

As may be noticed, the Ty/PtNP/GPH/Chit-CSPE biosensor has features similar or superior to various (bio)sensors used for Tyr detection.

### 3.7. Interference Study

To assess biosensor selectivity for Tyr detection in multicomponent samples, the influence of several chemical species, which might interfere with bioassay such as Ca^2+^, Mg^2+^ in the determination of 5 × 10^−5^ M Tyr under optimal experimental conditions was studied. The tolerance limit was calculated as being the maximum concentration of the interfering chemical species, which causes a relative error of ±5% for the quantitative determination of Tyr. The results obtained are shown in Table 2.

Tyr determination is influenced more strongly by the presence of ascorbic acid and uric acid, while Ca^2+^, Mg^2+^, Na^+^ have a reduced influence in quantitative determination. Considering these results, it may be argued that the biosensor developed in this study is selective for Tyr determination in multicomponent samples.

### 3.8. Repeatability and Stability of the Ty/PtNP/GPH/Chit-CSPE Biosensor

In order to analyse the repeatability values of Ty/PtNP/GPH/Chit-CSPE, the Tyr values were recorded by using the same biosensor and the square wave voltammetry in a 10^−5^ M Tyr solution. In between the measurements, the biosensor was removed from the assay solution, rinsed with 0.1 M PBS pH 7.0 and then stored for 5 min in PBS. The relative standard deviation for 10 successive measurements was 1.4%, which demonstrates that the biosensor may be used repeatedly for quantitative determinations.

The biosensor stability was analysed by recording the SWV response in the 10^−5^ M Tyr solution weekly for a four-week period. In between the measurements, the biosensor was stored in a refrigerator at 4 °C in a sealed closed box. Voltammetric results quantified as the anodic peak intensity proved that the biosensor retained 95.8% of the initial response, thus being almost unchanged four weeks after its manufacture. This very good stability may be related to the protective effect of the bionanocomposite matrix including graphene, chitosan and platinum nanoparticles, which prevents enzyme denaturation and loss of biocatalytic properties.

### 3.9. Use of the Biosensor in Bioassay and Validation of the Quantitative Determination Method

The final step in this study consisted in determining Tyr in biological (3 heparinized blood plasma samples—HBPS) and pharmaceutical samples by using the biosensor developed in this research study and in comparing the results obtained when using the standard determination method (absorbance at 275 nm). The results obtained are shown in Table 3.

As can be noticed, the differences between the values obtained for the analytical recovery are lower than 2% for all the samples analysed, which indicates that the biosensor developed in this research study is useful and may be used in bioassay. Analysis of variance (ANOVA) showed that there was no statistically significant difference at the 99% confidence level between the results obtained by using the two methods, i.e., the biosensor and the ultraviolet (UV) spectrometry at 275 nm [42]. In addition, the results obtained in the case of pharmaceutical samples were compared with the values of the active compound concentration mentioned on the label of commercial products, the almost identical values obtained demonstrating the accuracy of the biosensor measurements developed in this research study.

## 4. Conclusions

A new nanostructured enzyme biosensor was developed and characterized for the qualitative and quantitative determination of L-tyrosine from standard solutions and real samples. Tyr electro-oxidation at the biosensor detector element is favoured by using a biocompatible matrix to immobilize the enzyme and the nanomaterials, which provide a rapid transfer of electrons from the redox reaction. The biosensor has high sensitivity, low detection limit, and the interferences caused by other analytes are minimal. Repeatability and stability in Tyr determination are appropriate and recommend this biosensor for routine determinations. The analytical method involving the use of the biosensor and the SWV detection techniques was validated by comparing the results obtained when using this method with those obtained when using UV spectroscopy. The results confirmed that the method is reliable, accurate and applicable in the Tyr bioassay.

## Figures and Tables

**Table 1:** Comparison between the Ty/PtNP/GPH/Chit-CSPE biosensor and other (bio)sensors reported in literature for the detection of Tyr.

(Bio)Sensor	Detection Principle	Linear Range (M)	LOD (M)	Reference
Zeolite, CPE	DPV	1.2 × 10^−6^–90 × 10^−6^	3.2 × 10^−7^	[38]
MWCNT, GCE	HDV	9 × 10^−7^–3.5 × 10^−4^	3.5 × 10^−7^	[39]
B.P.Tyr/M/SN-MPTS/SPE	DPV	5 × 10^−8^–6 × 10^−4^	2 × 10−^8^	[8]
Fe-HA/Ty	Amperometry	1 × 10^−7^–1.1 × 10^−5^	2.45 × 10^−7^	[40]
EM/Ty	Optical	5 × 10^−6^–2 × 10^−4^	1 × 10^−6^	[41]
Ty/Chit/PtNP/GPH-CSPE	SWV	1 × 10^−7^–1 × 10^−4^	4.75 × 10^−8^	This work

DPV—differential pulse voltammetry; CPE—carbon paste electrode; MWCNT—multiwall carbon nanotubes; GCE—glassy carbon electrode; HDV—hydrodynamic voltammetry; B.P.Tyr—banana peel tissue tyrosinase; M—mediator; SN-MPTS—silicon nanoparticles with 3-mercaptopropyl trimethoxysilane groups; SPE—screen-printed electrode; Fe-HA—Fe-hydroxyapatite; EM—eggshell membrane.

**Table 2:** The interference of several chemical species on the quantitative determination of 10^−5^ M Tyr in optimal conditions.

Interfering Chemical Species	c/M	[Tyr]/M	RE (%)	Recovery (%)
Na^+^	0.1	4.98 × 10^−5^	−0.4	99.6
K^+^	0.1	5.04 × 10^−5^	0.8	100.8
Mg^2+^	0.1	4.95 × 10^−5^	−1	99
Ca^2+^	0.1	5.14 × 10^−5^	2.8	102.8
L-Lysine	0.01	5.15 × 10^−5^	3	103
L-Asparagine	0.01	5.22 × 10^−5^	4.4	104.4
Glycine	0.01	4.9 × 10^−5^	−2	98
L-Phenylalanine	0.01	4.94 × 10^−5^	−1.2	98.8
L-Ascorbic acid	0.001	5.45 × 10^−5^	9	109
Uric acid	0.001	5.44 × 10^−5^	8.8	108.8
d-Glucose	0.001	4.92 × 10^−5^	−1.6	98.4
L-Glutathione	0.001	5.25 × 10^−5^	5	105

c—concentration of interfering chemical species; RE—relative error in determination of Tyr.

**Table 3:** Quantitative determination of Tyr from real samples.

Sample	Method	[Tyr]/M Detected	[Tyr]/M Added	[Tyr]/M Found	Recovery (%)
L-Tyrosine (Solgar)	A	20 × 10^−6^	20 × 10^−6^	40.1 × 10^−6^	100.5
500 mg/capsule	B	18 × 10^−6^	20 × 10^−6^	38.2 × 10^−6^	101.0
L-Tyrosine (Solaray) 500 mg/capsule	A	30 × 10^−6^	20 × 10^−6^	49.9 × 10^−6^	99.5
	B	32 × 10^−6^	20 × 10^−6^	51.9 × 10^−6^	99.5
L-Tyrosine (Organika) 500 mg/capsule	A	25 × 10^−6^	20 × 10^−6^	45.1 × 10^−6^	100.5
	B	24 × 10^−6^	20 × 10^−6^	44.05 × 10^−5^	100.3
HBPS 1	A	54 × 10^−6^	50 × 10^−6^	104.2 × 10^−6^	100.4
	B	52 × 10^−6^	50 × 10^−6^	101.8 × 10^·6^	99.6
HBPS 2	A	78 × 10^−6^	50 × 10^−6^	127.9 × 10^−6^	99.8
	B	81 × 10^−6^	50 × 10^−6^	131.5 × 10^−6^	101.0
HBPS 3	A	95 × 10^−6^	50 × 10^−6^	145 × 10^−6^	100.0
	B	93 × 10^−6^	50 × 10^−6^	142.8 × 10^−6^	99.6

A—biosensor method; B—spectrophotometric method.
